# miR-30c-2-3p Regulates METTL14 Expression and Inhibits Cell Migration in Breast Cancer

**DOI:** 10.3390/cimb48060545

**Published:** 2026-05-23

**Authors:** Zeliha Emrence, Seyma Punar, Vahideh Zarerajabi, Sena Uslu, Neslihan Abaci, Sema Sirma Ekmekci

**Affiliations:** 1 Department of Genetics, Aziz Sancar Institute of Experimental Medicine, Istanbul University, 34093 Istanbul, Turkey; neslihan.abaci@istanbul.edu.tr (N.A.); sirmasem@istanbul.edu.tr (S.S.E.); 2 Department of Genetics, Institute of Graduate Studies in Health Sciences, Istanbul University, 34126 Istanbul, Turkey; seymapunar@ogr.iu.edu.tr (S.P.); vahideh.zarerajabi@ogr.iu.edu.tr (V.Z.); 3 Istanbul Faculty of Medicine, Istanbul University, 34093 Istanbul, Turkey; senauus@gmail.com

**Keywords:** miR-30c-2-3p, METTL14, m^6^A methylation, breast cancer, cell migration, epigenetic regulation

## Abstract

Breast cancer remains a leading cause of cancer-related mortality worldwide, with epigenetic mechanisms like N^6^ methyladenosine (m^6^A) modification playing a crucial role in tumorigenesis. The interaction between microRNAs and m^6^A regulators, such as the methyltransferase METTL14, is increasingly recognized as a key pathway in oncogenesis. This study investigated whether miR-30c-2-3p regulates METTL14 expression to influence global m^6^A levels and cell migration in breast epithelial (MCF12A) and breast cancer (MCF7) cell lines. Following transfection with miR-30c-2-3p mimics, successful overexpression was confirmed in both cell lines. Subsequent RT-qPCR and Western blotting analyses demonstrated that METTL14 mRNA and protein levels were significantly reduced at 24 and 48 h post-transfection (*p* < 0.05). Concurrently, global m^6^A RNA methylation levels decreased, with a more pronounced reduction observed in MCF12A cells (*p* < 0.001). Functionally, wound healing assays revealed that miR-30c-2-3p significantly inhibited migration, reducing wound closure by 30–44% in MCF7 cells and by 66–72% in MCF12A cells. These findings reveal a novel regulatory axis involving miR-30c-2-3p, METTL14, and m^6^A, suggesting that miR-30c-2-3p functions as a tumor suppressor and represents a promising biomarker and therapeutic target in breast cancer.

## 1. Introduction

Breast cancer (BC) remains one of the leading causes of cancer-related mortality among women worldwide, with its incidence continuing to rise globally [1]. It represents a highly heterogeneous disease at the molecular level and challenges such as recurrence, metastasis, and drug resistance are frequently encountered [2,3]. Therefore, elucidating the molecular mechanisms underlying the development and progression of breast cancer is crucial for identifying novel biomarkers that can aid in early diagnosis, therapeutic targeting, and evaluation of prognosis [4]. MicroRNAs (miRNAs) are endogenous, single-stranded, non-coding RNAs of 18–24 nucleotides that post-transcriptionally regulate gene expression by binding to the 3′ untranslated region (3′UTR) of target mRNAs, leading to translational inhibition or mRNA degradation [5]. Numerous studies have demonstrated that miRNAs play pivotal roles in various cancer types, functioning as either oncogenes or tumor suppressors [6,7]. Through these mechanisms, miRNAs modulate key cellular processes including proliferation, apoptosis, invasion, and metastasis. Among miRNA families, the miR-30 family—comprising miR 30a, -30b, -30c-1, -30c-2, -30d, and -30e—has been shown to act as a tumor suppressor in multiple cancers [8]. miR-30c, a member of this family, regulates essential cellular processes such as proliferation, differentiation, metabolism, and metastasis, and its reduced expression of this microRNA has been associated with poor prognosis [9,10]. In particular, increased miR-30c expression has been linked to improved response to tamoxifen therapy in estrogen receptor (ER)–positive breast cancer patients [7]. Conversely, miR-30c-2-3p expression has been reported to be downregulated in ER-negative breast cancers, where its overexpression suppresses cell migration and invasion by modulating the NF-κB signaling pathway and cell-cycle progression [11].

Epigenetic mechanisms, including histone modifications, DNA methylation, and RNA methylation, play crucial roles in regulating gene expression. Among RNA modifications, N6-methyladenosine (m^6^A) is the most prevalent internal modification in eukaryotic mRNAs [12,13]. m^6^A marks are dynamically regulated by methyltransferases (“writers,” such as METTL3, METTL14, and WTAP), demethylases (“erasers,” such as FTO and ALKBH5), and reader proteins that recognize and interpret these modifications [14]. Aberrant m^6^A modification has been implicated in tumor initiation and progression through its effects on mRNA stability, splicing, translation, and degradation [15]. Recent studies have demonstrated a bidirectional regulatory relationship between m^6^A modifications and non-coding RNAs, including miRNAs. While m^6^A influences miRNA biogenesis and maturation, miRNAs can also target m^6^A-related enzymes, thereby modulating m^6^A dynamics across the transcriptome [16,17]. This reciprocal regulation plays an important role in maintaining the balance of gene expression and has been implicated in cancer development.

In breast cancer, however, the role of METTL14—a core component of the m^6^A methyltransferase complex—remains controversial. Some studies have shown that METTL14 expression is downregulated in breast tumors, and that its overexpression suppresses cell proliferation, colony formation, and migration [18]. In contrast, other studies have reported elevated METTL14 levels that enhance cancer cell invasion and metastasis through m^6^A-dependent mechanisms [19]. Considering these conflicting findings, the potential role of the miR-30c-2-3p/METTL14 axis in breast cancer biology warrants further investigation.

MicroRNA-30c-2-3p (miR-30c-2-3p) is recognized as a regulatory miRNA involved in breast cancer biology, particularly through pathways associated with cell-cycle control and NF-κB signaling [11]. To explore its potential interaction with METTL14, we analyzed publicly available datasets, including GEPIA2 and the Human Protein Atlas, and observed alterations in METTL14 expression between breast cancer and normal tissues. Furthermore, bioinformatic prediction tools such as TargetScanHuman 8.0 and miRTarBase 9.0 identified a putative miR-30c-2-3p binding site within the METTL14 transcript, suggesting a possible regulatory relationship. Collectively, these findings provide preliminary evidence for a previously uncharacterized link between miR-30c-2-3p and m^6^A regulation via METTL14. Given that both miR-30c-2-3p and m^6^A modifications have been independently implicated in breast cancer, our study aimed to determine whether miR-30c-2-3p modulates m^6^A RNA methylation by targeting METTL14 and to characterize the downstream effects on cell migration in breast cancer and normal mammary epithelial cells.

## 2. Materials and Methods

### 2.1. Cell Culture

MCF7 (HTB-22) human breast adenocarcinoma and MCF12A (CRL-3598) non-tumorigenic mammary epithelial cell lines were obtained from the American Type Culture Collection (ATCC, Manassas, VA, USA). MCF7 cells were cultured in Dulbecco’s Modified Eagle Medium (DMEM) supplemented with 10% fetal bovine serum (FBS) and 1% penicillin–streptomycin (Pen/Strep). MCF12A cells were maintained in DMEM Ham’s F12 medium containing 10% FBS, 1% Pen/Strep, 20 ng/mL epidermal growth factor (EGF), and 0.5 µg/mL hydrocortisone. All cells were incubated at 37 °C in a humidified atmosphere containing 5% CO_2_.

### 2.2. miRNA Transfection

Cells were seeded into 6-well plates and allowed to adhere overnight before transfection. Transfections were performed using Lipofectamine RNAiMAX reagent (Thermo Fisher Scientific, Waltham, MA, USA) according to the manufacturer’s protocol, which is optimized for miRNA delivery. Cells were transfected with miR-30c-2-3p mimics (mirVana^®^ miRNA mimic (hsa-miR-30c-2-3p, Thermo Fisher Scientific (Carlsbad, CA, USA) to induce overexpression or with a negative control miRNA as a reference group. Following transfection, the cells were incubated for 24 h or 48 h, after which they were harvested for RNA isolation, protein extraction, or migration assays.

### 2.3. RNA Isolation and RT-qPCR

Total RNA was isolated from transfected and control cells using TRIzol reagent (Invitrogen, Carlsbad, CA, USA) based on the method originally described by Chomczynski and Sacchi (1987) [20]. The isolated RNA was dissolved in RNase-free water and stored at −80 °C until use. RNA concentration and purity were determined using a NanoDrop 2000 spectrophotometer (Thermo Scientific, Wilmington, DE, USA), and RNA integrity was verified by agarose gel electrophoresis.

For miRNA expression analysis, cDNA was synthesized using miRNA-specific stem-loop primers, whereas random hexamer primers were used for mRNA analysis. Quantitative real-time PCR (RT-qPCR) was carried out using SYBR Green Master Mix (SensiFAST, Bioline, London, UK). The RT-qPCR temperature cycling conditions used in this study are summarized in Table A1. U6 small nuclear RNA was used as the internal control for miRNA expression, and TATA-binding protein (TBP) was used for normalization of mRNA levels (Table A2). TBP and U6 expression levels showed no substantial variation across the experimental conditions evaluated in this study. Relative expression values were calculated using the ΔΔCt method, and all experiments were performed in biological triplicates.

### 2.4. Global m^6^A RNA Methylation Assay

Global m^6^A RNA methylation levels were determined using a colorimetric m^6^A RNA Methylation Quantification Kit (Epigentek, P-9005, Farmingdale, NY, USA) according to the manufacturer’s protocol. Total RNA samples were bound to assay wells, incubated with capture and detection antibodies, and treated with a colorimetric substrate. Absorbance was measured at 450 nm using a microplate reader. Relative m^6^A methylation levels were normalized to those of the control group.

### 2.5. Western Blot Analysis

Total proteins were extracted using RIPA buffer supplemented with protease and phosphatase inhibitors. Cell lysates were sonicated and centrifuged to remove debris, and the supernatants were collected. Protein concentrations were determined using the BCA Protein Assay Kit (Thermo Fisher Scientific, Waltham, MA, USA). Equal amounts of protein (30 µg) were separated by SDS–PAGE on 4–12% Bis-Tris gels (Invitrogen, Carlsbad, CA, USA) and transferred onto PVDF membranes using the iBlot Transfer Stack system (Invitrogen, Carlsbad, CA, USA). Membranes were blocked with 5% non-fat dry milk in TBS-T for 1 h at room temperature, followed by overnight incubation at 4 °C with primary antibodies against METTL14 (1:1000, Cell Signaling Technology, Danvers, MA, USA) and β-actin (1:5000, Sigma-Aldrich, Louis, MO, USA). After washing, membranes were incubated with HRP-conjugated secondary antibodies for 1 h at room temperature. Signals were visualized using enhanced chemiluminescence (WesternBright Sirius, Advansta, San Jose, CA, USA) and quantified with ImageJ software (version 1.54f; NIH, Bethesda, MD, USA).

### 2.6. Scratch Wound Healing Assay

To evaluate cell migration, a scratch wound healing assay was conducted. Cells were seeded in 6-well plates and cultured to 90–100% confluence. A sterile 200 µL pipette tip was used to create a linear scratch in the monolayer. Wells were gently washed with PBS to remove detached cells and then incubated in medium containing 1% FBS to minimize proliferation. Images were captured at 0, 24, and 48 h post-scratch using an inverted phase-contrast microscope. Wound areas were measured using ImageJ software, and migration rates were calculated as the percentage of wound closure relative to the initial wound area.

### 2.7. Statistical Analysis

All experiments were performed in biological triplicates. Data are expressed as mean ± standard error of the mean (SEM). Normality of data distribution was tested prior to analysis. Comparisons between two groups were performed using either an unpaired two-tailed Student’s *t*-test (for normally distributed data) or a Mann–Whitney U test (for non-parametric data). Statistical analyses were conducted using GraphPad Prism 8, and differences were considered statistically significant at *p* < 0.05.

## 3. Results

### 3.1. miR-30c-2-3p Expression in MCF7 and MCF12A Cells

To assess the efficiency of miR-30c-2-3p overexpression, MCF7 and MCF12A cell lines were transfected with miR-30c-2-3p mimics and incubated for 24 and 48 h. Total RNA was subsequently isolated and analyzed by real-time quantitative PCR (RT-qPCR). miR-30c-2-3p expression levels were markedly elevated compared to the corresponding controls. Specifically, in MCF7 cells, expression increased approximately 5145-fold at 24 h and 6706-fold at 48 h post-transfection. In MCF12A cells, miR-30c-2-3p levels increased by approximately 1362-fold at 24 h and 15514-fold at 48 h.

### 3.2. The Effect of miR-30c-2-3p on METTL14

**Figure 1:**
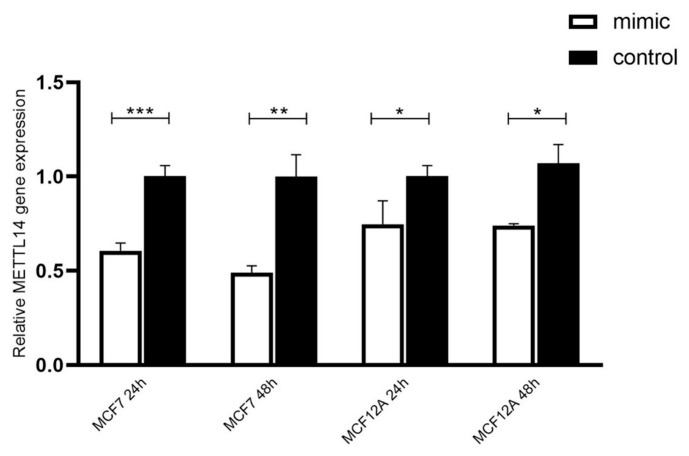
Relative expression of METTL14 mRNA levels in MCF7 and MCF12A cells. Relative expression of METTL14 mRNA levels in MCF7 and MCF12A cells transfected with miR-30c-2-3p mimic or negative control. Bars represent mean ± SEM (*n* = 3 per group). Control groups are shown as solid black bars, while miR-30c-2-3p mimic groups are shown as solid white bars. Expression levels were normalized to the control group within each condition. Statistical analysis was performed using unpaired two-tailed Student’s *t*-test. Significance levels are indicated as follows: * *p* < 0.05, ** *p* < 0.01, *** *p* < 0.001.

Following miR-30c-2-3p mimic transfection, the upregulation of miRNA expression was confirmed by RT-qPCR, and METTL14 mRNA levels were subsequently evaluated in MCF7 and MCF12A cell lines. In both cell types, a significant reduction in METTL14 expression was observed following mimic transfection. In MCF7 cells, METTL14 mRNA levels were significantly decreased at 24 h (*p* = 0.0006) and 48 h (*p* = 0.0019) compared to the control group. Similarly, in MCF12A cells, the reduction in METTL14 expression was also statistically significant at both 24 h (*p* = 0.0331) and 48 h (*p* = 0.0053) (Figure 1). Additional raw RT-qPCR data are provided in the Supplementary Data.

### 3.3. Expression of miR-30c-2-3p Reduces Global m^6^A RNA Methylation Levels

**Figure 2:**
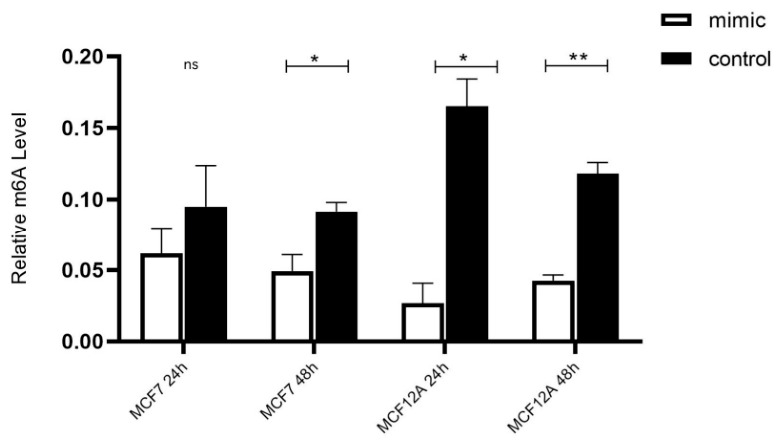
miR-30c-2-3p overexpression reduces global m^6^A RNA methylation levels. miR-30c-2-3p overexpression reduces global m^6^A RNA methylation levels in breast epithelial and cancer cell lines. Each condition was then compared to its respective negative control. Data are shown as the mean ± standard error of the mean (SEM) of three biological replicates. Asterisks indicate statistical significance between the control and mimic groups (* *p* < 0.05, ** *p* < 0.01; ns: not significant).

To investigate the regulation of global m^6^A RNA methylation by miR-30c-2-3p, MCF12A and MCF7 cell lines were transfected with miR-30c-2-3p mimics. Total RNA was isolated 24 and 48 h after transfection, after which global m^6^A levels were measured using a colorimetric assay kit. In MCF12A cells, expression of miR-30c-2-3p was associated with a significant decrease in global m^6^A levels compared to the control group after 24 and 48 h of incubation (*p* = 0.0009 and *p* = 0.0008, respectively). A decrease in m^6^A levels was also observed in MCF7 cells after mimic transfection. However, a statistically significant difference was only found at the 48 h time point (*p* = 0.0107), while the difference at the 24 h time point was not statistically significant (*p* = 0.1805) (Figure 2). Additional global m^6^A methylation data are provided in the Supplementary Data.

### 3.4. miR-30c-2-3p Reduces METTL14 Protein Expression

**Figure 3:**
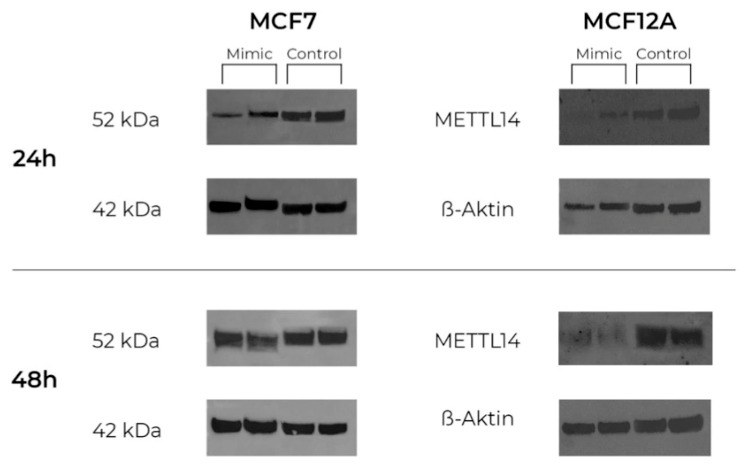
miR-30c-2-3p overexpression decreases METTL14 protein levels. miR-30c-2-3p overexpression decreases METTL14 protein levels in MCF7 and MCF12A cells. Western blot analysis showing METTL14 and β-actin (loading control) in MCF7 and MCF12A cell lines 24 and 48 h after transfection with miR-30c-2-3p mimics or negative control. In both cell lines, METTL14 protein levels were markedly reduced following miR-30c-2-3p overexpression.

To evaluate the effect of miR-30c-2-3p on METTL14 protein levels, Western blot analysis was performed in MCF7 and MCF12A cell lines following transfection with miR-30c-2-3p mimics. METTL14 and the loading control β-actin were analyzed at 24 and 48 h post-transfection. A reduction in METTL14 protein levels was observed in both cell lines upon miR-30c-2-3p overexpression compared to their respective controls. In MCF7 cells, this reduction was statistically significant at both 24 h (*p* = 0.032) and 48 h (*p* = 0.008). Similarly, in MCF12A cells, a statistically significant decrease was detected at 24 h (*p* = 0.045), which became more pronounced at 48 h (*p* = 0.011), (Figure 3).

### 3.5. miR-30c-2-3p Reduces Cell Migration

To evaluate the effect of miR-30c-2-3p on cell migration, a scratch wound healing assay was performed using MCF7 and MCF12A cell lines. Images were captured at 0, 24, and 48 h post-transfection, and wound closure was quantified using ImageJ software. In the control groups, progressive wound closure was observed over time in both cell lines, indicating active migration. In contrast, miR-30c-2-3p overexpression was associated with a reduction in wound closure, suggesting an inhibitory effect on cellular migration.

**Figure 4:**
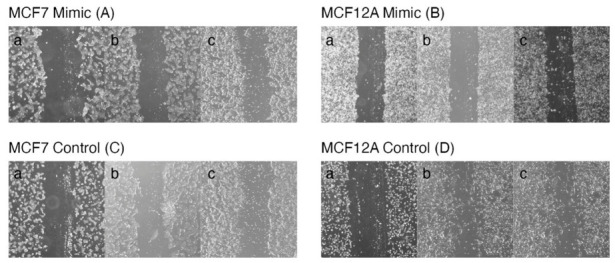
Effect of miR-30c-2-3p overexpression on cell migration in MCF7 and MCF12A cells. Scratch wound healing assay demonstrating the effect of miR-30c-2-3p overexpression on cell migration in MCF7 mimic (**A**), MCF12A mimic (**B**), MCF7 control (**C**), and MCF12A control (**D**) cells. Cells were photographed at 0 h (a), 24 h (b), and 48 h (c) post-transfection. In the control groups, progressive wound closure was observed over time, indicating active cell migration. In contrast, miR-30c-2-3p mimic transfection resulted in visibly reduced wound closure in both cell lines, showing its inhibitory effect on cellular migration.

In MCF7 cells, miR-30c-2-3p mimic transfection resulted in a 30% reduction in migration at 24 h and a 44% reduction at 48 h compared to control cells. The inhibitory effect was even more pronounced in MCF12A cells, with migration reduced by 66% at 24 h and 72% at 48 h. These results suggest that miR-30c-2-3p overexpression may impair the migratory capacity of both breast epithelial and breast cancer cell lines, with a stronger effect observed in the non-tumorigenic MCF12A cells (Figure 4). Additional raw migration assay data are provided in the Supplementary Data.

## 4. Discussion

Our study demonstrates that miR-30c-2-3p overexpression significantly downregulates METTL14 expression at both mRNA and protein levels, leading to a pronounced reduction in global m^6^A RNA methylation and impaired cell migration in both MCF7 and MCF12A cells. Notably, we observed a temporal difference in the reduction in global m^6^A levels between the two cell lines; while MCF12A cells exhibited a significant decrease as early as 24 h, MCF7 cells showed a significant reduction only at 48 h. This discrepancy may reflect differences in metabolic rates or epigenetic plasticity between non-tumorigenic and cancerous cells. Cancer cells often possess more robust compensatory mechanisms or altered RNA turnover rates, which might delay the manifestation of global methylation changes compared to normal epithelial cells. Collectively, these findings suggest that miR-30c-2-3p functions as a tumor suppressor in breast tissue, potentially by regulating METTL14-mediated m^6^A RNA methylation.

Members of the miR-30 family are known to regulate tumor progression by targeting genes involved in proliferation, differentiation, apoptosis, and invasion [6]. In particular, miR-30c-2-3p has been reported to inhibit NF-κB signaling and cell-cycle progression by downregulating TRADD and CCNE1 in estrogen receptor–negative breast cancer cells [11], consistent with our observation that its overexpression reduces migratory ability. Furthermore, decreased miR-30c-2-3p expression has been correlated with poor prognosis or inferior therapeutic response in breast cancer, supporting its role as a tumor suppressor [7,9].

Epigenetic RNA modifications such as N^6^-methyladenosine (m^6^A) are critical regulators of post-transcriptional gene expression [12,14]. METTL14, a core component of the m^6^A methyltransferase complex, functions with METTL3 and WTAP to catalyze adenosine methylation on mRNA. Dysregulation of m^6^A modification has been implicated in tumor initiation and progression through altered mRNA stability, translation, and degradation [14]. However, the biological role of METTL14 appears to be context-dependent across different cancer types. In hepatocellular carcinoma (HCC), for example, reduced METTL14 expression suppresses m^6^A-dependent microRNA maturation and promotes metastasis [21]. Likewise, colorectal cancer (CRC) models have shown that METTL14 downregulation facilitates epithelial-to-mesenchymal transition (EMT) and tumor progression [22,23]. Conversely, elevated METTL14 expression enhances proliferation and is associated with poor prognosis in pancreatic cancer and acute myeloid leukemia (AML) [24,25]. In breast cancer, findings remain contradictory. Dong et al. reported that METTL14 expression is significantly reduced in tumor tissues compared with normal controls and that its overexpression suppresses cell proliferation, colony formation, and migration [18]. In contrast, Yi et al. found that METTL14 expression was elevated in breast tumors and promoted invasion through m^6^A-dependent regulation of miR-146a-5p [19]. Our results are more consistent with the former model, as METTL14 levels decreased and migration was reduced following miR-30c-2-3p overexpression, suggesting that METTL14 suppression may be a downstream effect of miR-30c-2-3p activity.

Recent studies have highlighted a reciprocal regulatory relationship between miRNAs and the m^6^A machinery, where m^6^A influences miRNA biogenesis and miRNAs can, in this relationship, m^6^A influences miRNA biogenesis, and miRNAs can regulate the expression of m6A-related enzymes, including METTL3, METTL14, and FTO. [15,16]. Our findings are consistent with this bidirectional interaction and suggest that miR-30c-2-3p may act as an upstream regulator of METTL14 and m^6^A methylation dynamics in breast cancer.

In summary, we propose a miR-30c-2-3p/METTL14/m^6^A regulatory axis that could modulate both RNA methylation and cell migration in breast cancer. Considering the contrasting roles of METTL14 in different tumor types, this axis might represent a context-dependent epigenetic pathway in breast tissue. The ability of miR-30c-2-3p to influence both gene expression and RNA modification suggests that it could be further investigated as a potential diagnostic or prognostic biomarker and as a target for modulating m^6^A-related mechanisms in cancer.

## 5. Limitations of Study

This study was conducted using in vitro breast cell line models, which may not fully recapitulate the complexity of tumor behavior in vivo. Although we demonstrated that miR-30c-2-3p regulates METTL14 expression and affects cell migration, additional experiments using animal models or patient-derived samples would be necessary to confirm these findings in a physiological context. We also noted a substantial overexpression of miR-30c-2-3p, particularly in MCF12A cells (>13,000-fold); while this confirms successful transfection, we acknowledge that supraphysiological levels could potentially lead to non-specific off-target effects, although the consistent phenotypic results in both cell lines suggest a specific mechanism. Future studies using lower and more physiological levels of miR-30c-2-3p overexpression may further clarify whether the observed phenotypic effects persist under conditions that more closely reflect the in vivo cellular environment. Furthermore, to definitively confirm that the observed inhibition of migration is mediated exclusively through METTL14, future studies should include “rescue” experiments involving the overexpression of a METTL14 construct lacking the 3′UTR in miR-30c-2-3p-transfected cells. Finally, only limited downstream targets of METTL14 were examined; therefore, further studies are warranted to elucidate the broader impact of the miR-30c-2-3p–METTL14 interaction on m^6^A-dependent gene regulation.

## Data Availability

All data generated or analyzed during this study are included in this published article.

## Supplementary Materials

The following supporting information can be downloaded at: https://www.mdpi.com/article/10.3390/cimb48060545/s1.

## Abbreviations

The following abbreviations are used in this manuscript:

BC	Breast cancer
miRNA	microRNA
m^6^A	N6-methyladenosine
METTL14	Methyltransferase-like 14
METTL3	Methyltransferase-like 3
WTAP	Wilms tumor 1-associated protein
FTO	Fat mass and obesity-associated protein
ALKBH5	Alkylation repair homolog 5
3′UTR	3′ untranslated region
ER	Estrogen receptor
NF-κB	Nuclear factor kappa-light-chain-enhancer of activated B cells
EMT	Epithelial-to-mesenchymal transition

## Appendix A

### Appendix A.1

**Table A1:** Details of miRNA mimics, inhibitors, and transfection reagents used in this study.

REAGENT NAME	TYPE	MANUFACTURER	CATALOG NUMBER	SEQUENCE/NOTE
**MIRVANA® MIRNA MIMIC (HSA-MIR-30C-2-3P)**	miRNA Mimic	Thermo Fisher Scientific (Carlsbad, CA, USA)	4464066	5′-CUGGGAGAGGGUUGUUUACUCC-3′ (miRBase ID: MIMAT0000254)
**ANTI-MIR™ MIRNA INHIBITOR NEGATIVE CONTROL**	Negative Control	Thermo Fisher Scientific (Carlsbad, CA, USA)	AM17010	Commercial proprietary sequence (Scramble)
**LIPOFECTAMINE™ RNAIMAX TRANSFECTION REAGENT**	Transfection Reagent	Invitrogen (Thermo Fisher Scientific) (Carlsbad, CA, USA)	13778150	Optimized for miRNA delivery

### Appendix A.2

**Table A2:** List of primer sequences used for RT-qPCR analysis.

Gene	Primer Sequence	
	Forward 5′−3′	Reverse 5′−3′
METTL3	CTC TAT CCACCC ACA AGA	GAA CTG CTGGCT GTG CTG
FTO	GAG AGC GCGGCT AAG AAA	CAT GCT TGTAGT GTG AGA
METTL14	AGA AGT TACCGA CAG CTC	TAC GTT TTGTTT GGA CA
ALKBH5	TTC AAG CCTTCG GGT GTC	TGC TCA GGGTTT GTT TCC
WTAP	TTC CCA AGAGTT CGA TTG	CCT TGG TTGAGT CGC ATT
TATABP	ACT TGA CCT AAA GAC CAT TGC AC	CTT GAA GTC CAA GAA CTT AGC TGG
miR-30c-2-3p	AACACGTGTGTAAACATCCTACA	GTCGTATCCAGTGCAGGGT
U6	GCTTCGGCAGCACATATACTAAAAT	CGCTTCACGAATTTGCGTGTCAT
