# Supplementary material for: miR-30c-2-3p Regulates METTL14 Expression and Inhibits Cell Migration in Breast Cancer
Source: Curr Issues Mol Biol. 2026 May 23;48(6):545. doi: 10.3390/cimb48060545 (PMC13297355; doi:10.3390/cimb48060545)
Supplement: Supplementary file 1 [file cimb-48-00545-s001.zip › Supplementary_Data_3_Original_Blots .pdf]

Supplementary Data 3. Original uncropped Western blot images for METTL14 and β-actin with molecular weight markers.

The following supplementary material provides the original, uncropped Western blot images corresponding to Figure 3 in the manuscript.

Each blot shows the full membrane with all lanes visible. Lane 1 corresponds to the P7719 (NEB) protein size marker. Lanes 2 and 3 represent the mimic-transfected samples, and lanes 4 and 5 correspond to the control group samples. METTL14 and β-actin were detected on separate membranes run in parallel

All uncropped Western blot images are provided below in their original form. The file names indicate the corresponding figure number in the manuscript, the cell line, the target protein, the time point, and the uncropped status (e.g., Fig3_MCF7_METTL14_24h_uncropped).

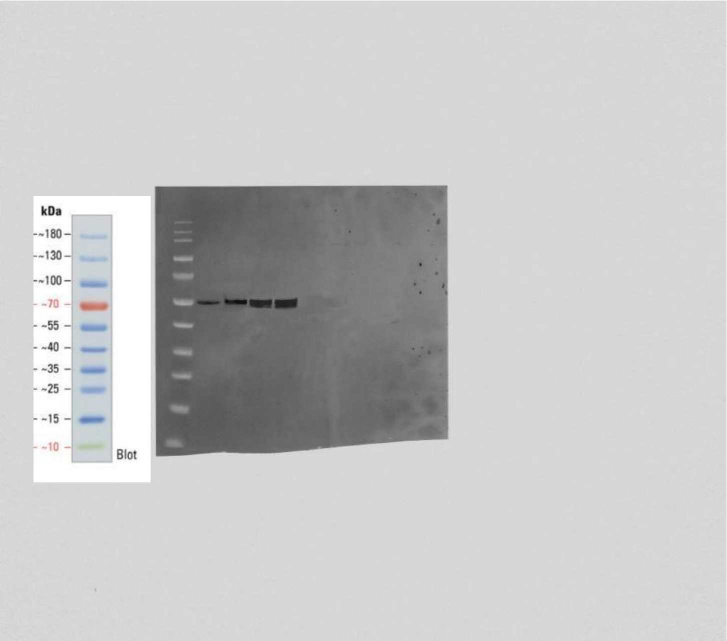

Fig3_mcf7_mettl14_24h_uncropped

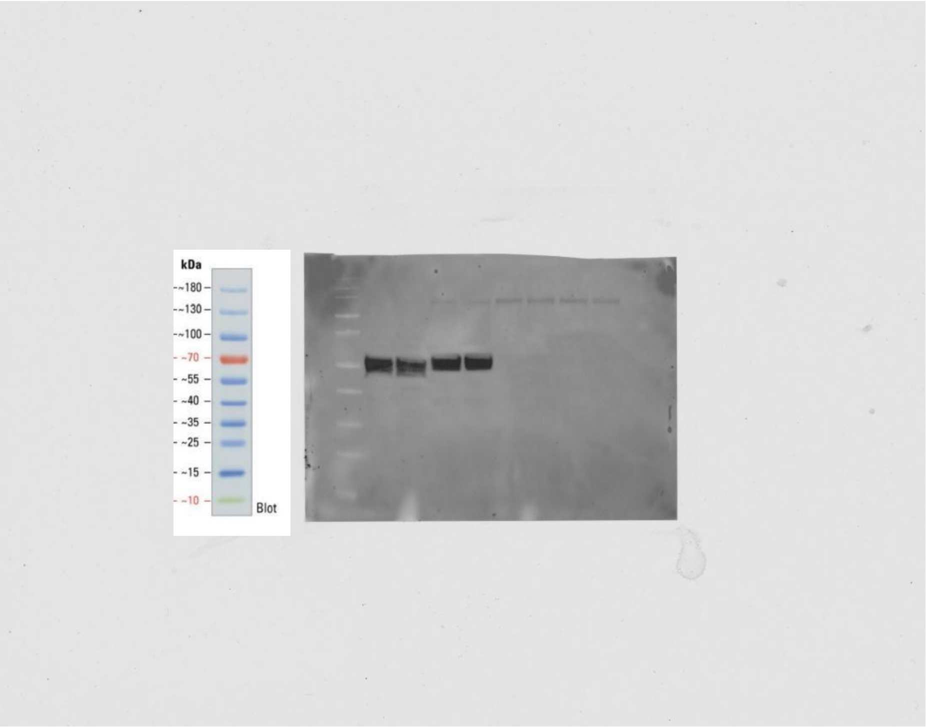

Fig3__mcf7_mettl14_48h_uncropped

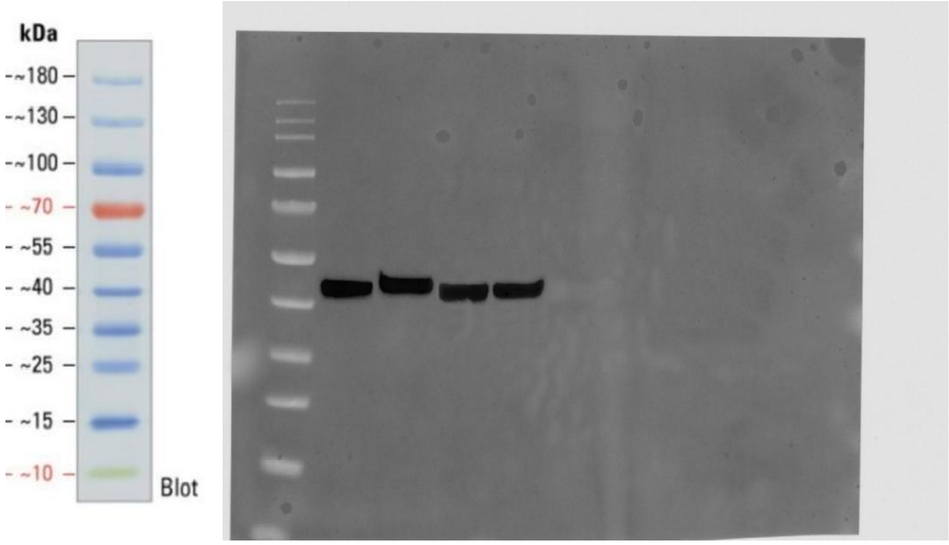

Fig3_mcf7_actin_24h_uncrooped .jpg

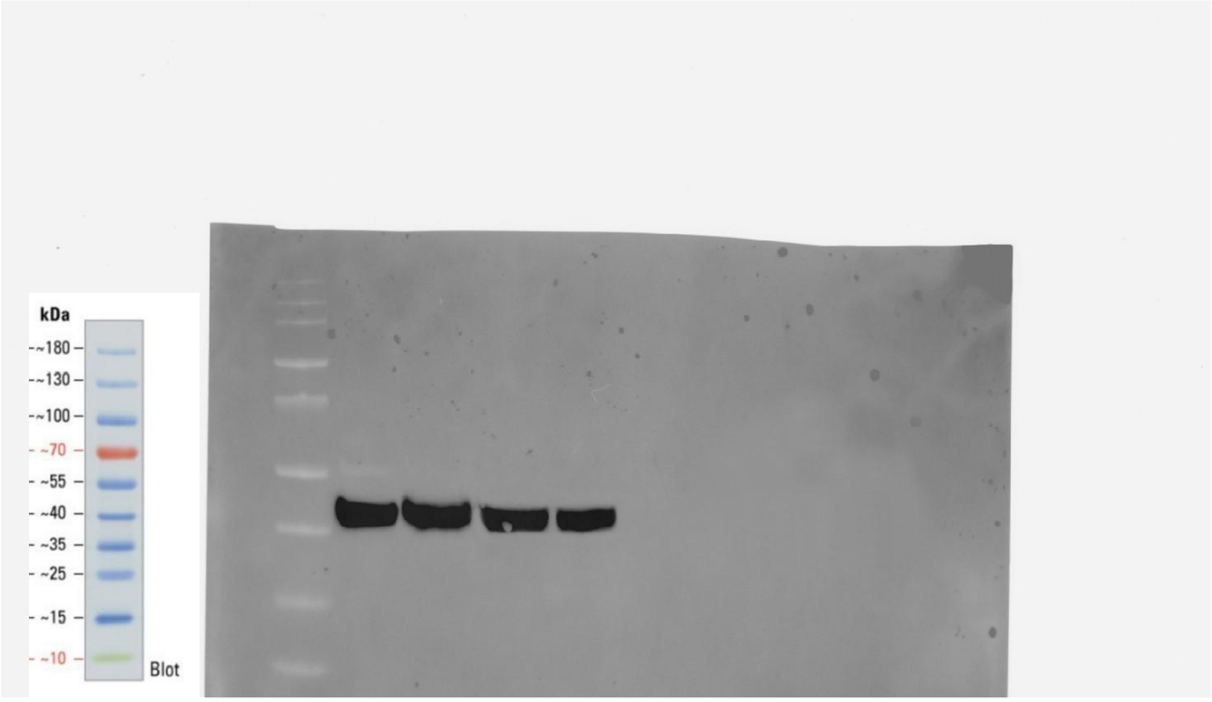

Fig3_mcf7_actin_48h_uncrooped .jpg

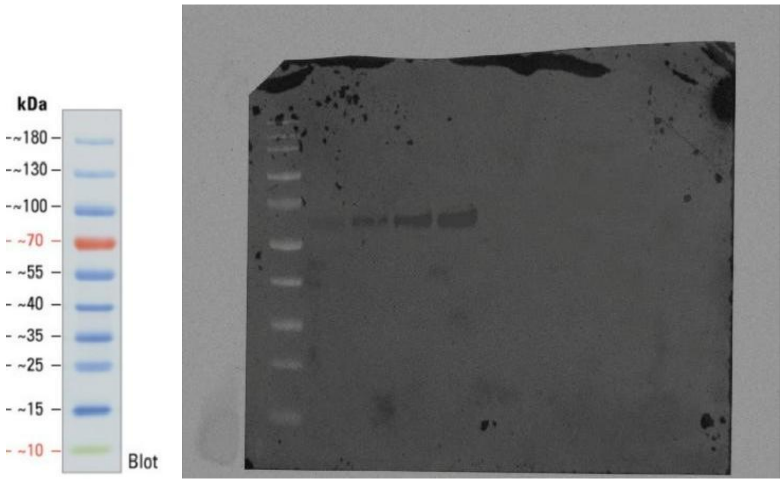

Fig3_mcf12a_mettl14_24h_uncropped.jpg

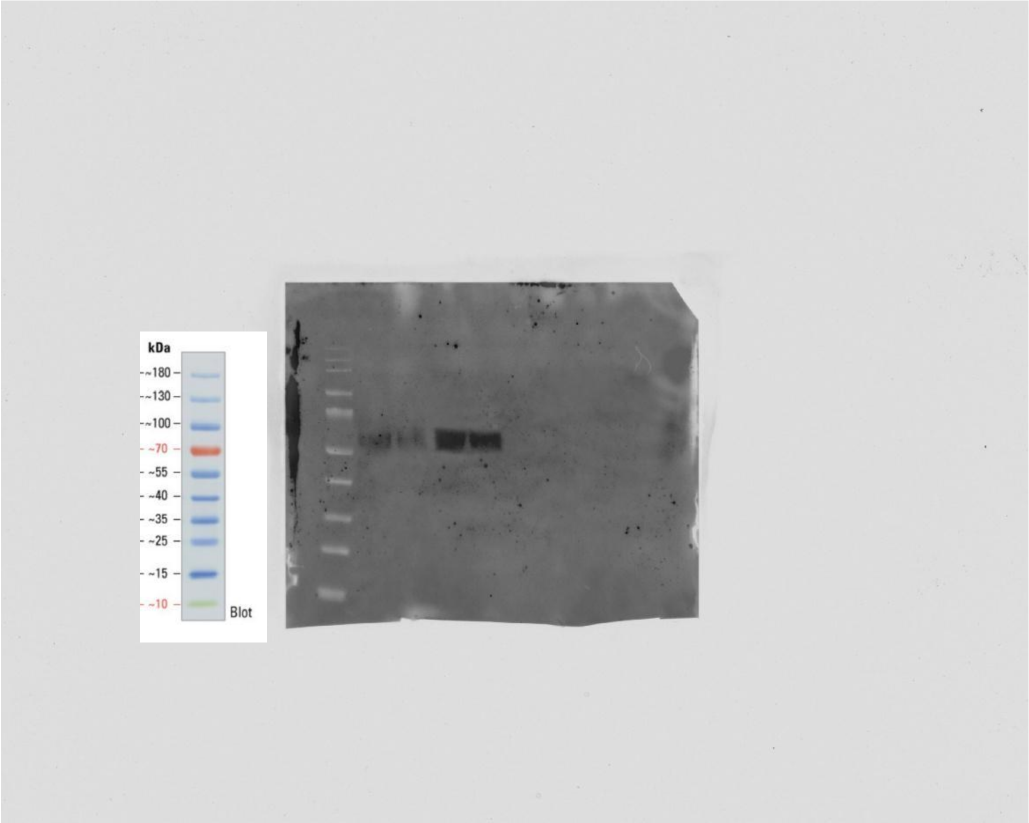

Fig3_mcf12a_mettl14_48h_uncropped.jpg

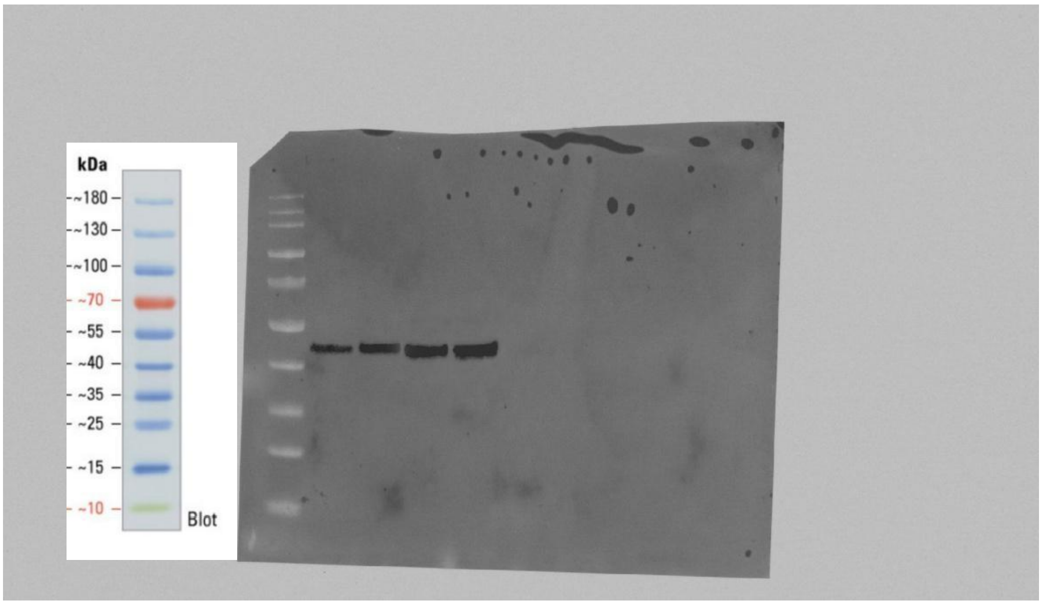

Fig3_mcf12a actin 24h_uncropped

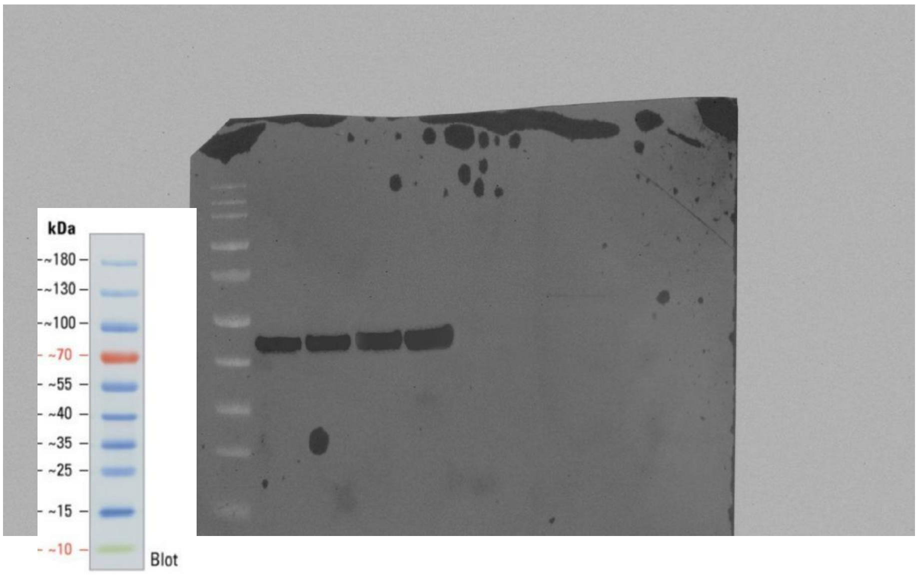

Fig3_mcf12a actin 48h_uncropped
